# The Effect of STDP Temporal Kernel Structure on the Learning Dynamics of Single Excitatory and Inhibitory Synapses

**DOI:** 10.1371/journal.pone.0101109

**Published:** 2014-07-07

**Authors:** Yotam Luz, Maoz Shamir

**Affiliations:** 1 Department of Physiology and Cell Biology, Faculty of Health Sciences Ben-Gurion University of the Negev, Beer-Sheva, Israel; 2 Department of Physics, Faculty of Natural Sciences Ben-Gurion University of the Negev, Beer-Sheva, Israel; Plymouth University, United Kingdom

## Abstract

Spike-Timing Dependent Plasticity (STDP) is characterized by a wide range of temporal kernels. However, much of the theoretical work has focused on a specific kernel – the “temporally asymmetric Hebbian” learning rules. Previous studies linked excitatory STDP to positive feedback that can account for the emergence of response selectivity. Inhibitory plasticity was associated with negative feedback that can balance the excitatory and inhibitory inputs. Here we study the possible computational role of the temporal structure of the STDP. We represent the STDP as a superposition of two processes: potentiation and depression. This allows us to model a wide range of experimentally observed STDP kernels, from Hebbian to anti-Hebbian, by varying a single parameter. We investigate STDP dynamics of a single excitatory or inhibitory synapse in purely feed-forward architecture. We derive a mean-field-Fokker-Planck dynamics for the synaptic weight and analyze the effect of STDP structure on the fixed points of the mean field dynamics. We find a phase transition along the Hebbian to anti-Hebbian parameter from a phase that is characterized by a unimodal distribution of the synaptic weight, in which the STDP dynamics is governed by negative feedback, to a phase with positive feedback characterized by a bimodal distribution. The critical point of this transition depends on general properties of the STDP dynamics and not on the fine details. Namely, the dynamics is affected by the pre-post correlations only via a single number that quantifies its overlap with the STDP kernel. We find that by manipulating the STDP temporal kernel, negative feedback can be induced in excitatory synapses and positive feedback in inhibitory. Moreover, there is an exact symmetry between inhibitory and excitatory plasticity, i.e., for every STDP rule of inhibitory synapse there exists an STDP rule for excitatory synapse, such that their dynamics is identical.

## Introduction

**Figure 1:**
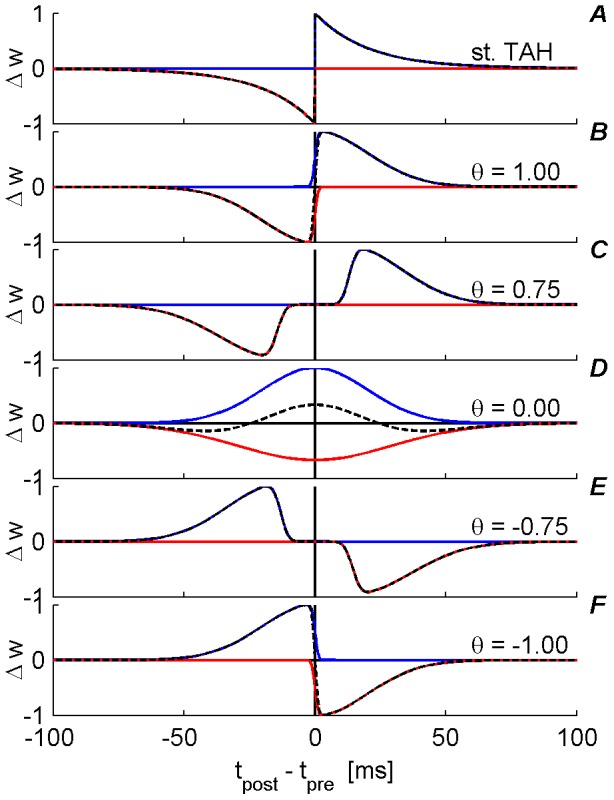
Illustration of different STDP temporal kernels (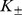) as defined by equations (7) and (8) with the “standard exponential TAH” as a reference. Each plot (normalized to a maximal value of 1 in the LTP branch) qualitatively corresponds to some experimental data. In all plots, the blue curve represents the potentiation branch 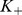, the red curve represents the depression branch 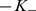 and the dashed black curve represents the superposition/sum of 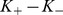. For simplicity, all plots were drawn with the same 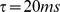. (A) The “standard exponential TAH” [1], [18]. (B) 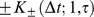 Alternate approximation to the standard exponential TAH [1], . (C) 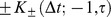 Temporally asymmetric Anti-Hebbian STDP [15]. (D) 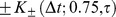 TAH variation [12], [19]. (E) 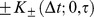 Temporally symmetric Hebbian STDP [16], [17]. (F) 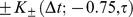 Variation to a temporally asymmetric Anti-Hebbian STDP [19]

Spike timing dependent plasticity (STDP) is a generalization of the celebrated Hebb postulate that “neurons that fire together wire together” to the temporal domain, according to the temporal order of the presynaptic and postsynaptic spike times. A temporally asymmetric Hebbian (TAH) plasticity rule has been reported in experimental STDP studies of excitatory synapses [1]–[3], in which an excitatory synapse undergoes long-term potentiation when presynaptic firing precedes the postsynaptic firing and long-term depression is induced when the temporal firing order is reversed, e.g., Figure 1A.

Many theoretical studies [4]–[9] that followed these experiments used an exponentially decaying function to represent the temporal structure of the STDP. Throughout this paper we term this STDP pattern the “standard exponential TAH”. Gütig and colleagues [7] also provided a convenient mathematical description for the dependence of STDP on the synaptic weight in the standard exponential TAH STDP rule:
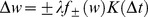
(1)

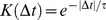
(2)

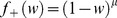
(3)

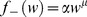
(4)where 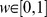 is the dynamic parameter that describes the synaptic strength; 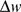 is the modification of 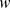 following pre (−) or post (+) synaptic firing; 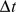 is the time difference between the presynaptic and postsynaptic firing; 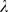 is the learning rate; 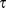 is the temporal decay constant and 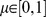 and 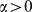 are dimensionless parameters of the model that characterize the weight dependent component of the STDP rule. This representation introduces a convenient separation of variables, in which the synaptic update is given as a product of two functions. One function is the temporal kernel of the STDP rule, i.e. 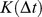, and the other is the weight dependent STDP component, i.e. 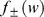. For convenience, throughout this paper we shall adopt the notation of Gütig and colleagues for the weight dependence of the STDP rule, 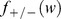, equations (3) – (4). This function, 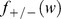, is characterized by two parameters: the relative strength of depression – 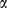, and the degree of non-linearity in 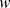 of the learning rule – 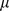. Note, that other choices for 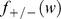 have also been used in the past [5],[10],[11].

### Properties of the “standard exponential TAH”

As previously shown [6], [7], the standard exponential TAH model can generate positive feedback that induces bi-stability in the learning dynamics of an excitatory synapse. For a qualitative intuition into this phenomenon, consider the case of a weight-independent STDP rule, also termed the additive model, i.e., 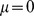. If the synaptic weight is sufficiently strong, there is a relatively high probability that a presynaptic spike will be followed by a postsynaptic spike. Hence, causal events (i.e., 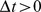 post firing after pre) are more likely to occur than a-causal events (with 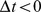). Because the STDP rule of the standard exponential TAH model implies LTP for 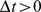 there is a greater likelihood for LTP than for LTD. Thus, a “strong” synapse will tend to become stronger. On the other hand, if the synaptic weight is sufficiently weak, then pre and post firing will be approximately uncorrelated. As a result, the stochastic learning dynamics will randomly sample the area under the STDP temporal kernel. Here we need to consider two types of parameter settings. If the area under the causal branch in equation (1) is larger than the area under the a-causal branch, 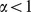, the net effect is LTP for weak synapses as well. Thus, in this case, all synapses will potentiate until they reach their upper saturation bound at 1. Hence, the regime of 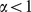, in this case, is not interesting. On the other hand, if the area under the a-causal branch is larger than the area under the causal branch, 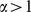, random sampling of the STDP temporal kernel by the stochastic learning dynamics (in the limit of weakly correlated pre-post firing, mentioned above) will result in LTD. Thus, in the interesting regime, 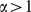, a “weak” synapse will tend to become weaker; thus producing the positive feedback mechanism that can generate bi-stability.

It was further shown [7] that this positive feedback can be weakened by introducing the weight dependent STDP component via the non-linearity parameter 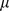 in equations (3) and (4). Setting 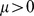 decreases the potentiation close to the upper saturation bound and decreases the depression close to the lower saturation bound; thus, for sufficiently large values of 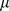 the learning dynamics will lose its bi-stability.

Experimental studies have found that the temporally asymmetric Hebbian rule is not limited to excitatory synapses and has been reported in inhibitory synapses as well [12]. Similar reasoning shows that in the case of inhibitory synapses the standard exponential TAH induces negative feedback to the STDP dynamics. It was shown [13] that this negative feedback acts as a homeostatic mechanism that can balance feed-forward inhibitory and excitatory inputs. Interestingly, Vogels and colleagues [14] studied a temporally symmetric STDP rule for inhibitory synapses, and reported that this type of plasticity rule also results in negative feedback that can balance the feed-forward excitation. This raises the question whether inhibitory plasticity always results in a negative feedback regardless of the temporal structure of the STDP rule? On the other hand, theoretical studies have shown that the inherent positive feedback of excitatory STDP causes the learned excitatory weights to be sensitive to the correlation structure of the pre-synaptic excitatory population – for different choices of STDP rules [5], [7], [10]. Does STDP dynamics of excitatory synapses always characterized by a positive feedback?

### Outline

**Figure 2:**
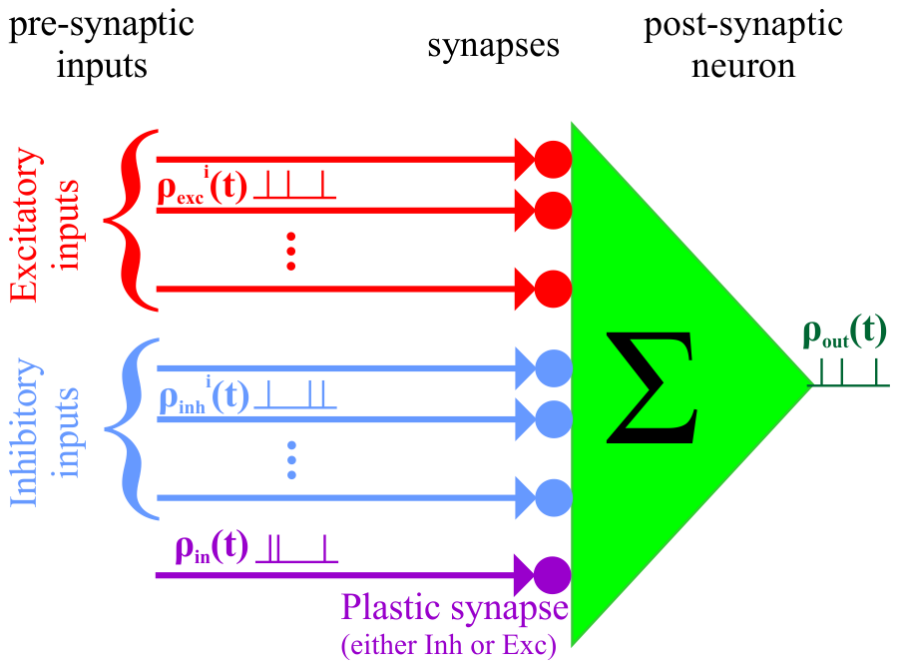
Model architecture. The STDP dynamics of a single either excitatory or inhibitory synapse is studied in purely feed-forward model. In all of the simulations presented here, the activity of the presynaptic inputs is modeled by a homogeneous Poisson process, with mean firing rate 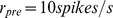. The synaptic weights of all synapses except one is kept fixed at a value of 0.5. The post synaptic neuron is simulated using an integrate and fire model as elaborated. See Methods for further details.

Although theoretical research has emphasized the standard exponential model, empirical findings report a wide range of temporal kernels for both excitatory and inhibitory STDP; e.g., [1], [12], [15]–[19], (see also the comprehensive reviews by Caporale and Dan [20] and Vogels and colleagues [21]). Here we study the effect of the temporal structure of the STDP kernel on the resultant synaptic weight for both excitatory and inhibitory synapses. This is done in the framework of learning of a single synapse in a purely feed-forward architecture, as depicted in Figure 2. First, we suggest a useful STDP model that qualitatively captures these diverse empirical findings. Below we define our STDP model. This model serves to study a large family of STDP learning rules. We derive a mean field Fokker-Planck approximation to the learning dynamics and show that it is governed by two global constants that characterize the STDP temporal kernel. Stability analysis of the mean-field solution reveals that the STDP temporal kernels can be classified into two distinct types: Class-I, which is always mono-stable, and Class-II that can bifurcate to bi-stability. Finally, we discuss the symmetry between inhibitory and excitatory STDP dynamics.

## Results

### Generalization of the STDP rule

In order to analyze various families of STDP temporal kernels found in experimental studies [1], [12], [15]–[19] we represent the STDP as the sum of two independent processes: one for potentiation and the other for depression. The synaptic update rule that we use throughout this paper is given by:
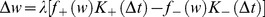
(5)


Note that the main distinction between equation (5) and (1), is that here, equation (5), the 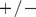 signs denote potentiation and depression, respectively; while in equation (1) the 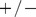 signs denote causal/a-causal branch. Thus, in our model for every 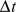 the synapse is affected by both potentiation and depression; whereas, according to the model of equation (1) the synapse undergoes either potentiation or depression – depending on the sign of 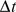. For example, in the standard exponential TAH model defined above in equation (2), this generalization implies a 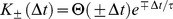 temporal kernel, where 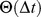 is the Heaviside step function. For the weight dependent STDP component (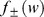) we adopt the formalism of equations (3) and (4). Below we describe a workable parameterization for the STDP temporal kernel. 

### Skew-Normal kernel

Here we used the Skew-Normal distribution function to fit the temporal kernels of the STDP rule, 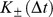. Note that the specific choice of the Skew-Normal distribution is arbitrary and is not critical for the analysis below. Other types of functions may serve as well. The “Skew-Normal distribution” is defined by:
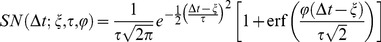
(6)where 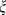 is the temporal shift, 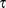 is the temporal decay constant, and 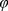 is a dimensionless constant that affects the skewness of the curve and 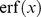 is the Gaussian error function. It is also useful to reduce the number of parameters that define the STDP temporal kernel. Thus, we define:



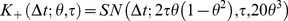
(7)

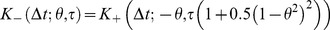
(8)where 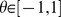 is a single continuous dimensionless parameter of the model that characterizes the STDP temporal kernel and 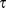 is the time constant of the exponential decay of the potentiation branch. The mapping of 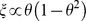 ensures that the temporal shift parameter, 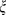, will be zero for 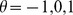. In order to obtain temporally symmetric Mexican hat STDP rule for 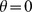 one needs to demand 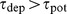, where 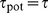 and 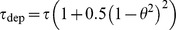. We also required 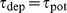 for 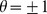, in order to be compatible with several previous studies. This reduction in parameters was chosen in order to capture the qualitative characteristics of various experimental data; however, other choices are also possible. Figure 1B-F illustrates how one can shift continuously from a temporally asymmetric Hebbian kernel (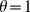,Figure 1B) to a temporally asymmetric anti-Hebbian kernel (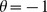,Figure 1F). Figure 1A shows the temporal kernel of the standard exponential TAH model, compare with 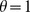, Figure 1B.

### “Mean field” Fokker-Planck approximation

We study the STDP dynamics of a single feed-forward synapse to a postsynaptic cell receiving other feed-forward inputs through synapses that are not plastic. We assume that all inputs to the cell obey Poisson process statistics with constant mean firing rate, 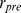; that the presynaptic firing of the studied synapse is uncorrelated with all other inputs to the postsynaptic neuron; and that the synaptic coupling of a single synapse is sufficiently weak. The STDP dynamics is governed by two factors: the STDP rule and the pre-post correlations. To define the dynamics one needs to describe how the pre-post correlations depend on the dynamical variable, 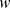. Under the above conditions one may assume that the contribution of a single pre-synaptic neuron that is uncorrelated with the rest of the feed-forward input to the post-synaptic neuron will be small. Thus, it is reasonable to approximate the pre-post correlation function (see Methods – equation (26)) up to a first order in the synaptic strength 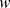 (e.g., [8], [22]–[24]), yielding:



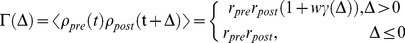
(9)where 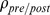 is the instantaneous firing of the pre/post synaptic cell represented by a train of delta functions at the neuron's spike times (see Methods), 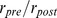 is the pre/post synaptic mean firing rate; and the function 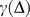 describes the change in the conditional mean firing rate of the postsynaptic neuron at time 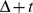 following a presynaptic spike at time 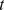. Note that we use upper case 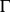 to represent the full pre-post correlations, 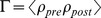, whereas 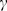 denotes the first order term in the synaptic weight, 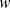, of these correlations.

In the limit of a slow learning rate, 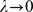, one obtains the mean-field Fokker-Planck approximation to the stochastic STDP dynamic (see Methods – equation (27)), and using the linear approximation of the pre-post correlations, equation (9), yields:
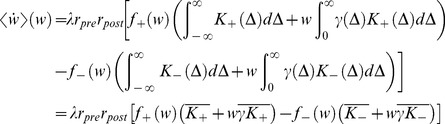
(10)where 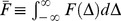 denotes the mean over time (using equation (9) with 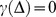 for 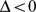).

In our choice of parameterization, 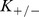 are set to have the same integral; i.e., 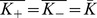. The difference between the strength of potentiation and depression of the STDP rule is controlled by the parameter 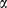 (equation (4)). Substituting expressions (3) & (4) into equation (10) yields:
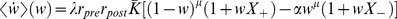
(11)where 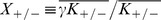 are constants that govern the mean-field dynamics. A fixed point solution, 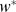, of the mean-field Fokker-Planck dynamics, 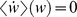, satisfies:



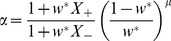
(12)


### Numerical simulations – the steady state of STDP learning

We performed a series of numerical simulations to test the approximation of the analytical result of the mean-field approximation at the limit of vanishing learning rate, using a conductance based integrate-and-fire postsynaptic neuron with Poisson feed-forward inputs (see Methods for details; a complete software package generating all the numerical results in this manuscript can be downloaded as File S1). We simulate a single postsynaptic neuron receiving feed-forward input from a population of 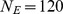 excitatory neurons and 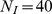 inhibitory neurons firing independently according to a homogeneous Poisson process with rate 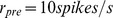. All synapses except one (either excitatory or inhibitory) were set at a constant strength (of 0.5). The initial conditions for the plastic synapse were as specified bellow.

**Figure 3:**
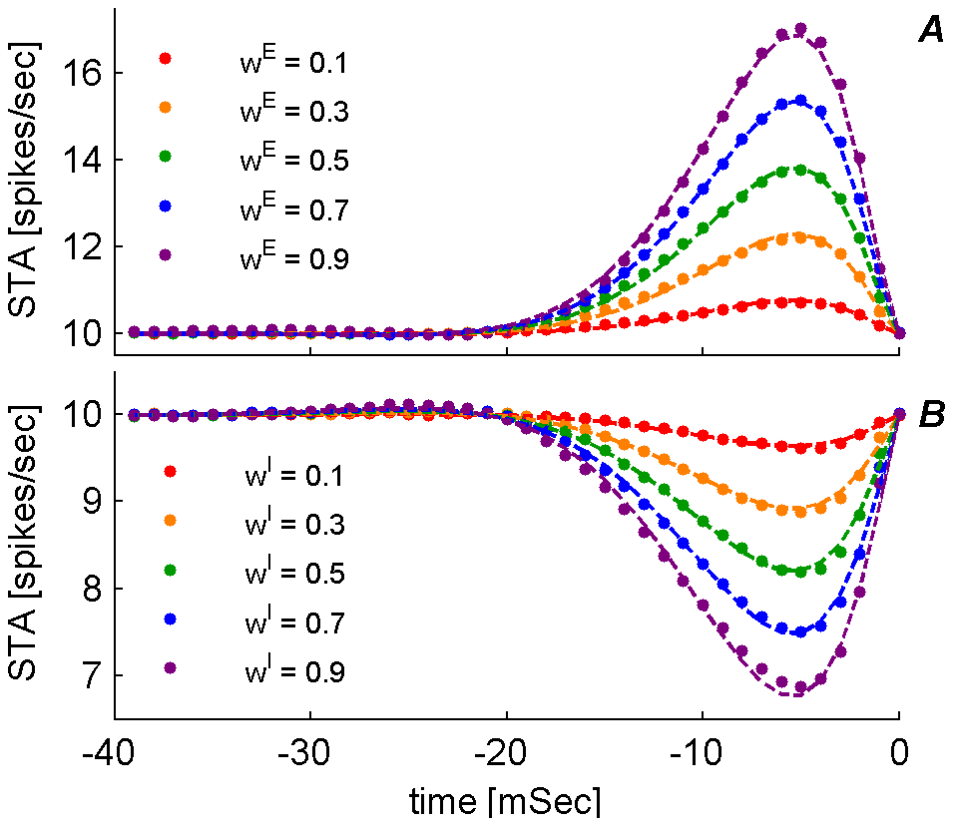
Spike Triggered Average (STA) of a single presynaptic input. The conditional mean firing rate of the presynaptic cell given that the postsynaptic cell has fired at time 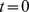, is plotted as function of time. (A) Excitatory synapse (B) Inhibitory synapse. Each set of dots (color coded) is the conditional mean firing rate calculated over 1000 hours of simulation time with fixed synaptic weights and presynaptic firing rates on all inputs. The different sets correspond to a different presynaptic weight (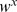) on a single synapse on which the STA was measured. The respective dashed lines show the numerical fitting of the form 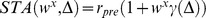 where 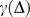 takes the revised formula: 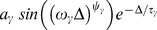. For every type of synapse, i.e., excitatory (in A) and inhibitory (in B), the parameters describing 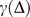, namely 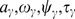, were chosen to minimize the least square difference between the analytic expression and the numerical estimation of the STA. These parameters were then used to calculate 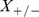.

We first estimated the spike triggered average (STA) firing rate of a single presynaptic neuron triggered on postsynaptic firing, in order to approximate the function 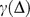, equation (9). Figure 3 shows the STAs of excitatory (**A**) and inhibitory (**B**) synapses for varying levels of synaptic weights (color coded), as were estimated numerically (dots). The dashed lines show smooth curve fits to the STA. The specific *temporal* structure of these curves depends upon particular details of the neuronal model. Nevertheless, the linear dependence on the synaptic weight is generic for weak synapses; thus, in line with the assumed linearity of the model, equation (9). The STA shows the conditional mean firing rate of the pre-synaptic neuron, given that the post fired at time 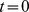. In the limit of weak coupling, 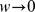, pre and post firing are statistically independent and the conditional mean equals the mean firing rate of the pre, 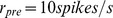. For an excitatory synapse, as the synaptic weight is increased the probability of a post spike following pre will also increase. Consequently, so will the likelihood of finding a pre spike during a certain time interval preceding a post spike. Hence, the STA of an excitatory synapse is expected to show higher amplitude for stronger synapse (as shown in **A**). Correspondingly, the STA of an inhibitory synapse is expected to show a more negative amplitude for stronger synapse (as shown in **B**).

To fit the STA with an analytic function we used 
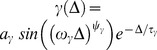
, with the fitted parameters 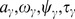 for both the inhibitory and excitatory cases. This ad hoc approximation serves to enable the numerical integration that calculates the constants 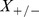 that govern equation (11) for the mean field approximation to the STDP dynamics.

**Figure 4:**
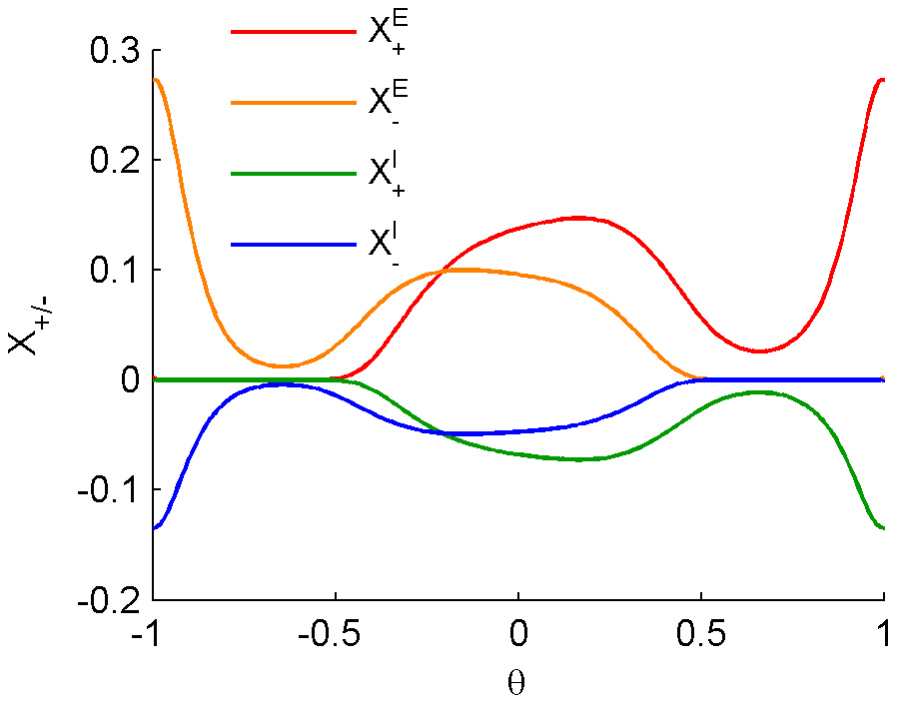
Mean field constants 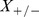 of equation (11) for the excitatory and inhibitory synapses of the neuronal model used in our numerical simulations, as a function of 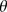. These values were calculated using numerical integration (see File S1) with 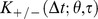 as defined by equations (7) and (8), with 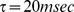 as set throughout the simulations, and with the fitted formula for 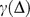.

All the richness of physiological details that characterize the response of the post-synaptic neuron affect the STDP dynamics only via the two constants 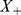 and 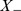. These two constants 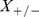 denote the overlap between the temporal structure of the pre-post correlations, 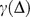, and the temporal kernel, 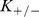, of the potentiation/depression kernel, respectively, Figure 4. Consequently, as 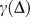, is positive for excitatory synapses and negative for inhibitory synapses – so are the constants 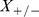. In addition, as the correlations in our model are causal, 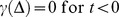, the constant 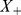 (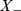) is expected to decay to zero when the STDP kernel 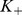 (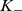) vanishes from the causal branch, 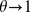 (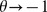). For the specific choice of parameters in our simulations, 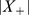 obtains its maximal value at 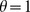. However, one may imagine other choice of parameters in which 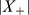 will obtain its maximal value at 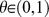. Note, from Figure 4, that the crossing of the 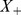 and 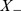 curves, is coincidentally almost the same for both synapse types, and is obtained at 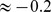. The significance of this point is discussed below.

### Fixed point solutions for the STDP dynamics

**Figure 5:**
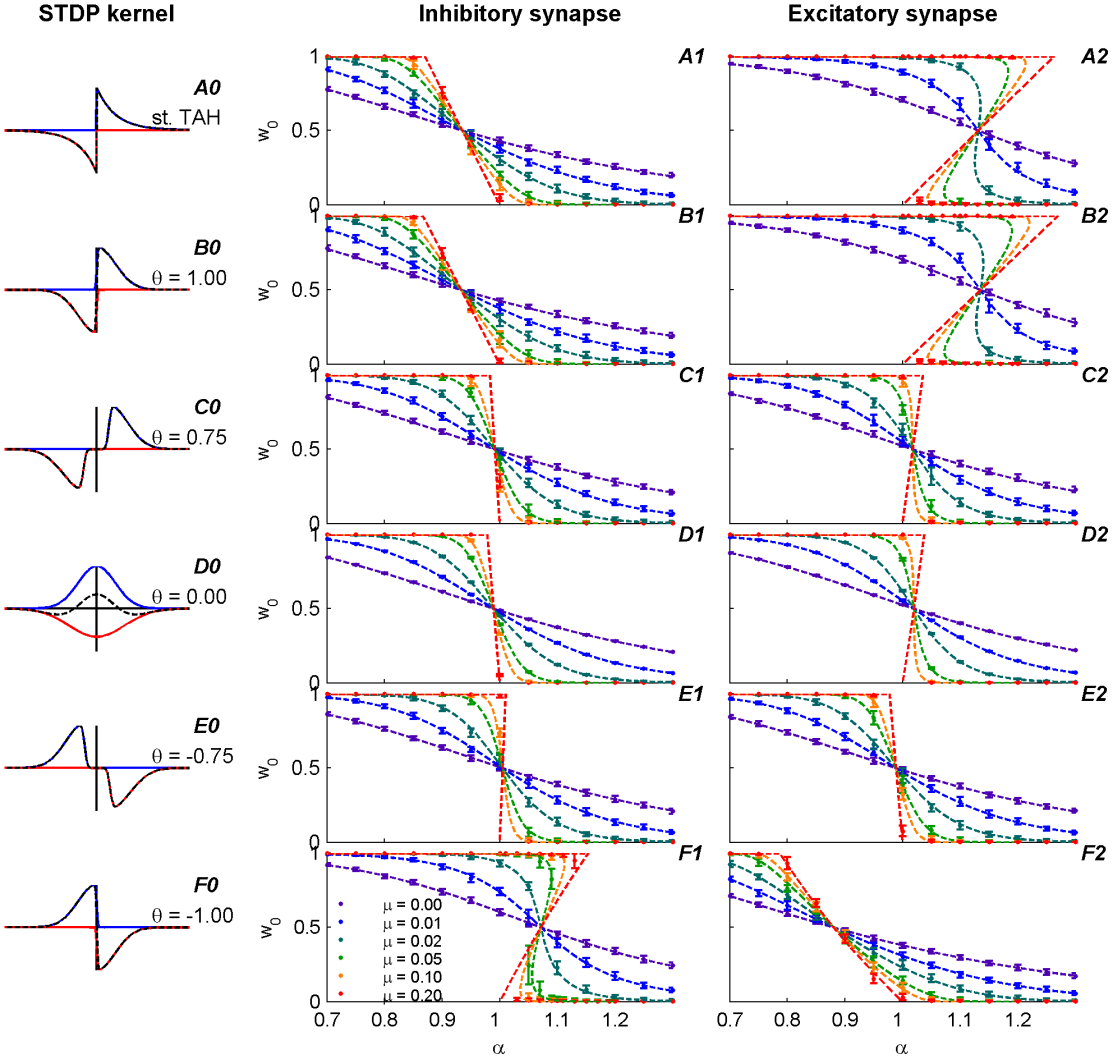
The fixed point solution (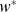) of equation (12) (dotted lines), is compared to the asymptotic synaptic weight (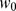) (circles), of a single synapse learning dynamics for various learning rules as defined by equation (5). Each of the panels in the middle column (for inhibitory synapse) and in the right column (for excitatory synapse) explores the weight dependent STDP component, 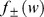 of equations (3) and (4), for representative set of 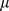 (shown by different colors as depicted in the legend) as a function of 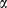. The different rows correspond to different STDP kernels, 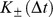 as shown by the panels in the left column. The circles and error bars represent the mean and standard deviation of the synaptic weight (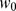), calculated over the trailing 50% of each learning dynamics simulation (see Methods). The mean field constants {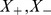} were numerically calculated using the 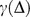 constants estimated as in Figure 3. The dotted lines were computed by equation (12) that was calculated for 10,000 sequential values of 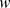 in 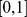. To this end, we replaced 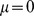 with 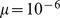 in order to use equation (12) to plot the dashed red line. Initial conditions for the simulations: for the majority of the simulations we have simply used 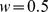 as initial condition for the plastic synaptic weight. In order to show the bi-stable solutions in panels (A2, B2, F1), for 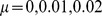 and 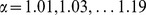 we ran two simulations one with initial condition 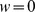 and another with initial condition 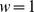. (A0-F0) are the STDP kernels (as in Figure 1) used in the simulations. (A1-F1) results for the inhibitory synapse simulations. (A2-F2) results for the excitatory synapse simulations.

Figure 5 shows 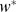 as a function of 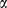 for different values of 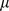 (color coded, note that 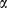 and 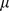 are the parameters that characterize the synaptic weight dependence of the STDP rule, equations (3) and (4)). The panels depict different STDP setups that differ in terms of the temporal kernels as well as the type of synapse (excitatory/inhibitory). These two factors affect the mean field equations via 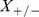. The dashed lines show the solution to the fixed point equation, equation (12), using the numerically calculated 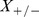. The fixed points were also estimated numerically by directly simulating the STDP dynamics in a conductance based integrate and fire neuron (circles and error bars).

For the estimation of the steady state value of the synaptic weight, the simulations were set to run for 5 hours of simulation time, which, according to manual offline analyses of convergence time scales, is much more than twice the time required for the system to converge and fluctuate around its steady state. The circles and error bars depict the mean ± standard deviation of the synaptic weight, as estimated from the last 2.5 hours of the simulation (weights were recorded at a 1 Hz sample rate). Note the high agreement between the fixed point solution (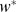) of equation (12), and the asymptotic synaptic weight (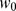) as estimated by the numerical simulation (regression coefficient of 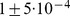 with 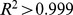 when performing a regression test on the entire set {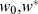} presented in each of the panels).

The panels of Figures 5A and 5B compare the standard exponential TAH rule of equation (2), in A, and our current STDP model with 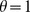 in B, for a representative set of parameters {(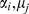)} applied to the examined synapse (middle column for inhibitory synapse and right column for excitatory synapse). Note that some lines may overlap each other near the boundaries: 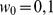. As is evident from the figures, the results of the two models coincide. In particular, the Hebbian STDP dynamics of inhibitory synapses is characterized by a one to one function 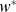 of 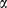 and there is no bi-stability, as previously reported [13], [14]. On the other hand, the Hebbian STDP of excitatory synapses is characterized by bi-stable solutions at low levels of 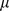 below a certain critical value, see e.g., [6], [7]. Thus, the current model with 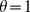 coincides with previous results.

The panels of Figure 5F show the results of a temporally asymmetric *Anti*-Hebbian STDP with 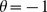. In striking contrast to the Hebbian STDP, in this case, inhibitory plasticity is characterized by bi-stability whereas, the excitatory plasticity is characterized by mono-stability.

The panels of Figures 5C and 5E explore two other types of asymmetric rules (Hebbian and *Anti*-Hebbian respectively). These results show similar behavior as 5B and 5F in terms of the classification of STDP kernels discussed in the next section.

The panels of Figure 5D show the results of the symmetric STDP with 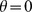 – note, that the dynamics of inhibitory synapse under the symmetric STDP rule, is characterized by a one to one function 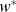 of 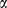 corresponding to negative feedback, as previously reported [14].

### Stability of the fixed point solution

The stability of the fixed point solution 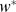 to equation (12) is determined by the sign of the partial derivative of the dynamical equation, equation (11), with respect to the synaptic weight:
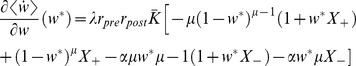
(13)


On the other hand, examination of Figure 5 suggests that the stability of the fixed point is governed by the sign of 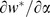. Taking the logarithm and the derivative with respect to 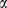 of both sides of equation (12), one obtains:
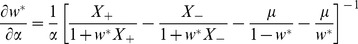
(14)

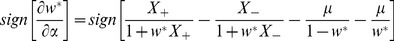
(15)where the last equality holds as 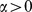. At the fixed point, substituting equation (12) into equation (13) one obtains:



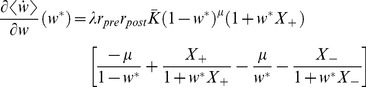
(16)Yielding:



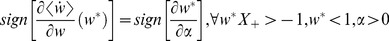
(17)


Hence, for 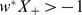, the fixed point solution in Figure 5 is stable in segments with negative slope, and unstable in segments with positive slope. Note that in our simulation setup 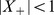 (cf. Figure 4); thus, the condition 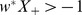 holds for all values of 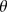 in our case.

Revisiting the different scenarios depicted in Figure 5, we note the existence of two qualitatively different behaviors; namely, one that can only show mono-stability (A, C, and F) and the other has the potential for bi-stability (in panels B, D, and E). We use this behavior to classify the different STDP temporal kernels that are parameterized by the single variable 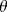. We shall term “class-I temporal kernels” the temporal kernels such that 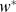 is mono-stable for all 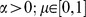. We shall term “class-II temporal kernels” the temporal kernels such that 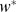 is bi-stable for some 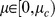 and some 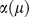. Note that this classification depends on the type of synapse (which via 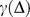 together with 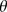 determine 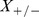). In addition, we note the existence of a special solution at 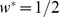 that is invariant to 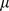, and enables us to obtain a simple condition for this classification. In class-I kernels the derivative 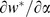 at 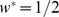 is always negative, whereas in class-II models there is a critical value of 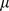 below which the derivative changes its sign.

### The “μ-invariant” solution and the critical μ

As 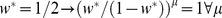, the solution of the fixed point equation, equation (12), at 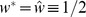 is 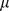-invariant. For a given STDP temporal kernel (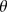), i.e. a given set of {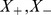} (see Figure 4; and note that 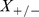 are also determined by the pre-post correlation structure via 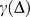), the solution of 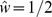 is obtained with:
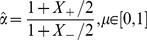
(18)


Substituting the 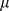-invariant solution, equation (18), into equation (15), yields



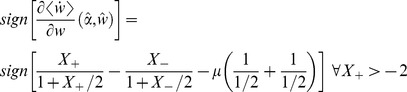
(19)


Thus, the condition for instability of the 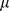-invariant solution is:
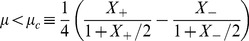
(20)


Thus, for 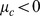 the 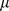-invariant solution, 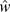, is stable for all values of 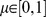 and the STDP rule is class-I for that synapse. On the other hand, if 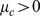 the STDP rule is class-II. This classification depends solely on the values of {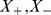}. In our simulation setup 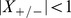 (see Figure 4), thus the classification of the parameter combinations is simply determined by the sign of (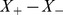); i.e. the manifold that is determined by the condition {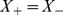} separates the parameter space (that characterizes the STDP rule and the synapse) between class-I and class-II.

### Bimodal distribution near 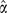


**Figure 6:**
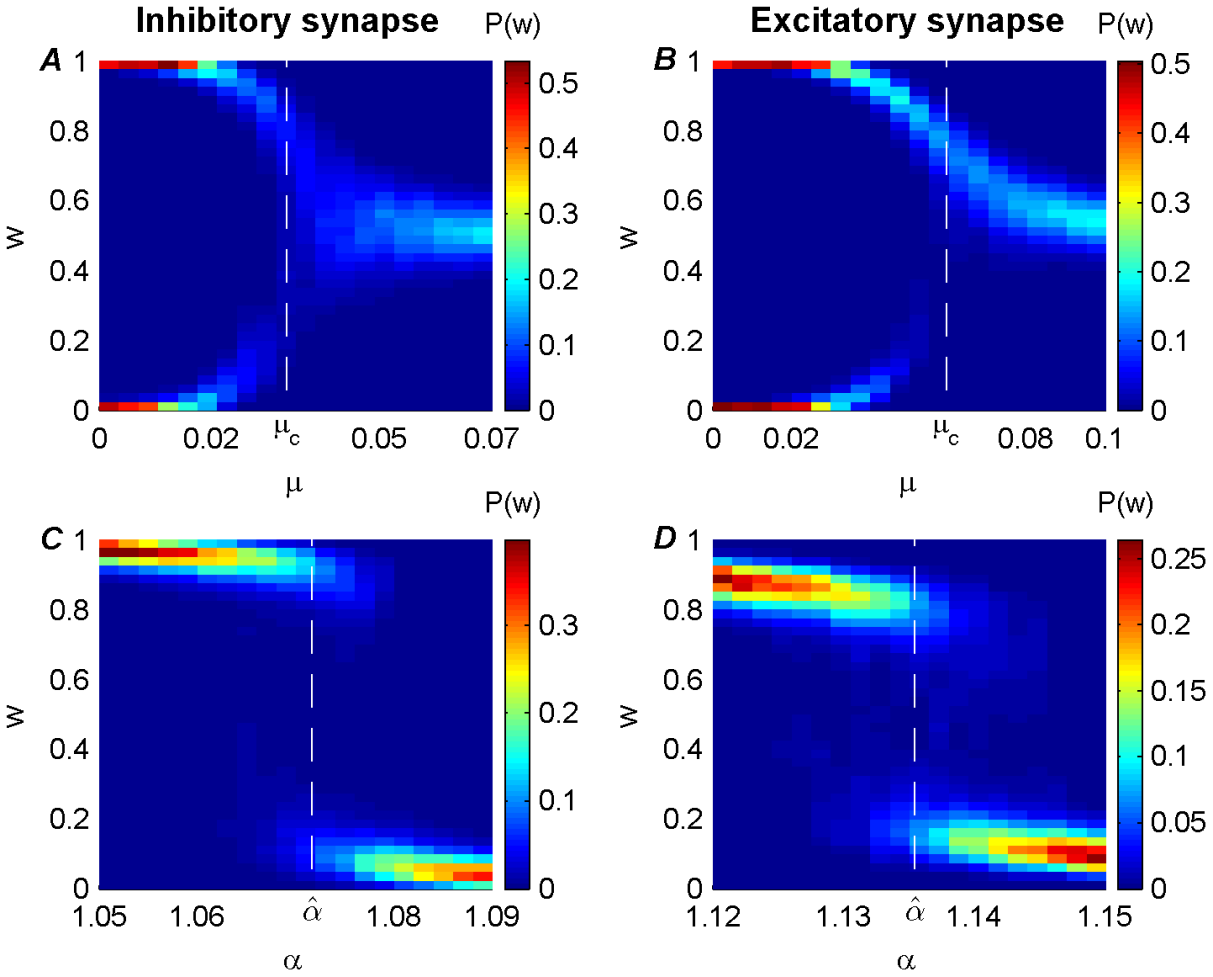
Bifurcation plots along the two parameters (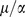) of the weight dependent STDP component, 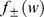 (see equations (3) and (4)) near 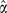 of equation (18). Panels display the synaptic weight distribution (color coded) for the various parameter setups: (A) Inhibitory synapse with anti-Hebbian (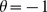, see also Figure 1F) rule, with fixed 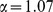 and varied 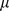. (B) Inhibitory synapse with anti-Hebbian (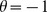, see also Figure 1F) rule, with fixed 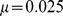 and varied 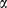. (C) Excitatory synapse with Hebbian (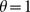, see also Figure 1B) rule, with fixed 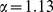 and varied 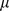. (D) Excitatory synapse with Hebbian (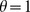, see also Figure 1B) rule, with fixed 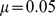 and varied 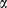. The dashed white line marks 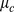 in A and B, and 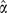 in C and D.

Figure 6 depicts (using numerical simulations with set of class-II parameters) the bifurcation plots for the learning dynamics for inhibitory (A, B) and excitatory (C, D) synapses. For inhibitory synapses the anti-Hebbian (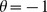) plasticity rules were chosen, and for the excitatory synapses, the Hebbian (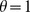). The panels show the resultant distribution of the synaptic weight color-coded after 21×101 of 5 hours of simulations for 21 values of the bifurcating argument (either 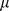 or 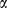) along the abscissa. In order to calculate the synaptic weight distribution for the set of parameters without the bias of initial conditions, 101 simulations were performed with different initial weight values evenly spaced from 0 to 1. The rationale for running the simulations for 5 hours each was to make sure that the learning dynamics had reached a steady state regime and the synaptic weight fluctuated around it for the entire trailing 2.5 simulation hours. During these trailing 2.5 simulation hours, the synaptic weights were recorded at a 1 Hz sample rate. For the estimation of the weight distribution, all the samples from the 101 simulations (differing only by their initial conditions) were used with 40 evenly spread bins between 0 and 1.

As expected from the analysis, there was a bifurcation along the 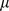 dimension (top panels), in which above 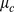 the distribution was uni-modal whereas below 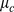 the distribution was bi-modal. Along the 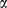 dimension (bottom panels) the distribution resembled the theoretical (dashed) curves of Figure 5 (without the unstable segment of 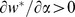).

### Symmetry and phase transition along θ

The high degree of similarity between the simulation results for inhibitory and excitatory synapses (Figure 5) stems from the fact that they obey the same mean-field equation (11), albeit with a different set of parameters. Thus, an excitatory synapse, 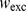, with a specific choice of parameters {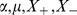} obeys the exact same mean-field equation as (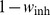), where 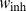 is an inhibitory synapse with the transformed set of parameters 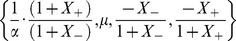 and a somewhat different learning constant (note that 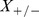 are positive for excitatory synapses and negative for inhibitory ones, see Figure 4).

**Figure 7:**
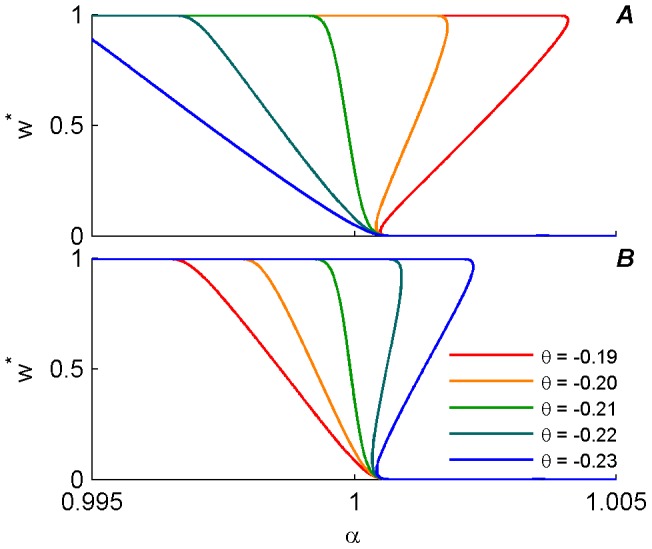
Fixed point solution, 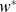, of the mean field approximation, (plotted using equation (12)), as a function of 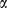, at 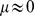 is shown for different values of 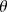 (color coded). Using 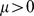 yields continuity of the curves at the extreme values (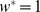 and 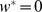), which makes the picture clearer. On the other hand as the value of 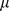 increases the unstable regime of 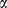 gets smaller and the resolution for 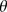 steps plotted should decrease. Thus, to plot these lines, we used 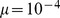 which is sufficiently close to 0 to illustrate the phase transition with high accuracy in 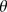. (A) Excitatory synapse. (B) Inhibitory synapse

This symmetry is illustrated for different STDP temporal kernels in Figure 7, where the mean field fixed point, 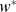, is plotted as a function of 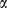 for different values of 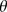 (color coded) at 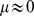. The different 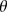 were chosen around 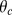 which is defined by the condition 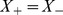 (see Figure 4) to display the phase transition from class-I to class-II along this parameter. Coincidentally, in our simulations and the chosen model (equations (7) and (8)), this specific 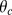 was almost the same for excitatory and inhibitory synapses; i.e. for both synapses 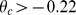 and 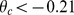
(see Figure 4). Under these conditions, for an excitatory synapse, 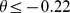 defines the class-I kernels, and 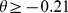 the class-II, whereas for an inhibitory synapse, 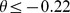 defines the class-II kernels, and 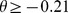 the class-I.

## Discussion

The computational role of the temporal kernel of STDP has been studied in the past. Câteau and Fukai [8] provided a robust Fokker-Planck derivation and analyzed the effects of the structure of the STDP temporal kernel. However, their analysis focused on excitatory synapses and the additive learning rule (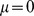). Previous studies have linked the Hebbian STDP of inhibition with negative feedback which acts as a homeostatic mechanism that balances the excitatory input to the postsynaptic cell [13], [14]. Positive feedback and bi-stability of STDP dynamics have been reported only for excitation, and linked to sensitivity to the input correlation structure [6], [7]. Here it was shown that the STDP of both excitation and inhibition can produce either positive or negative feedback depending on the parameters of the STDP model. Thus, for example, it was reported that both a temporally asymmetric Hebbian STDP (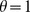) and a temporally symmetric learning rule (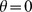) for inhibitory synapses generate negative feedback [13], [14]. These reports are in-line with our finding that the critical 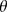 for transition from negative to positive feedback for inhibition is negative (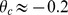).

In general, STDP dynamics of single synapses was classified here into two distinct types. With class-I temporal kernels, the dynamics is characterized by a negative feedback and has a single stable fixed point. In contrast, class-II temporal kernels are characterized by a sub-parameter regime in which the system is bi-stable (has positive feedback), and another sub-parameter regime with negative feedback. However, the mechanism that generates the negative feedback, (i.e., the stabilizing mechanism) in the two classes is different in nature. Whereas in class-I the negative feedback is governed by the convolution of the pre-post correlations with the temporal kernel, (i.e. the mean field constants 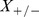, similar to the homeostatic mechanism in [13]), in class-II, the stabilizing mechanism is the non-linear weight dependent STDP component, 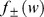. Hence, there is no reason a-priori to assume that the negative feedback in class-II should act as a homeostatic mechanism.

We found that there is no qualitative difference between the STDP of excitatory and inhibitory synapses and that both can exhibit class-I and class-II dynamics. Moreover, there is an exact symmetry between the excitatory and inhibitory STDP under a specific mapping of the parameters {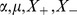}. This symmetry results from the fact that the mean-field dynamics depend solely on the global mean field constants 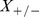. It is important to note that although neural dynamics is rich and diverse, due to the separation of time scales in our problem, the STDP dynamics only depends on these fine details via the global mean field constants 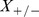.

Certain extensions to our work can be easily implemented into our model without altering the formalism. For example, empirical studies report different time constants for depression and potentiation, e.g. [1]. However, although in our simulations we used identical time constants at 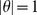, for 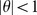 the depression time constant is larger than the potentiation time constant in our simulations. Moreover, our analytical theory depends on the time constants only via 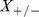. Consequently, changing time constants or any other manipulation to the temporal kernel can be incorporated into our mean-field theory by modifying 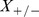. Similarly, assuming separation of time-scales between short term and long term plasticity, the effect of short term plasticity can be incorporated by modifying 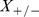 accordingly.

STDP has also been reported to vary with the dendritic location, e.g. [18], [25]. For a single synapse this effect can also be modeled by a modification of the parameters 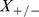. However, the importance of the dendritic dependence of STDP may reside in the interaction with other plastic synapses along different locations on the dendrite. Network dynamics of a 'population' of plastic synapses is beyond the scope of the current paper and will be addressed elsewhere.

In our model we assumed that the contribution of different "STDP events" (i.e., pre-post spike pairs) to the plastic synapse are summed linearly over all pairs of pre and post spikes, see e.g. equation (21). However, empirical findings suggest that this assumption is a mere simplification, and that STDP depends on pairing frequency as well as triplets of spike time and bursts of activity, e.g. [3], [26]–[30]. The computational implications of these and other non-linear interaction of spike pairs in the learning rule, as well as the incorporation of non-trivial temporal structure into the correlations of the pre-synaptic inputs to the cell are beyond scope of the current paper.

Empirical studies have reported a high variability of STDP temporal kernels over different brain regions, locations on the dendrite and experimental conditions, e.g., [1], [12], [15], [17]–[19]. Here we represented the STDP rule as the sum of two separate processes, one for potentiation and one for depression with an additional parameter, 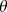, that allows us to continuously modify the temporal kernel and qualitatively obtain a wide spectrum of reported data. Representation of STDP by two processes has been suggested in the past. Graupner and Brunel [31], for example, proposed a model for synaptic plasticity in which the two processes (long term potentiation and depression) are controlled by calcium level. Thus, in their model the control parameter is a dynamical variable that may alter the plasticity rule in response to varying conditions. In our work, however, we did not model the dynamics of 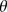. Moreover, we assumed that 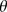 remains constant during timescales that are relevant for synaptic plasticity. It is, nevertheless, tempting to speculate on a metaplasticity process [32], [33] in which the temporal structure of the STDP rule is not hard wired and can be controlled and modified by the central nervous system. Thus, in addition to controlling the learning rate, 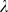, or the relative strength of potentiation-depression, 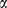, a metaplasticity rule may affect the learning process by modifying the degree of 'Hebbianitty', 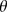. Such a hypothesis, if true, may account for the wide range of STDP kernels reported in the experimental literature. How can such a hypothesis be probed? One option for addressing this issue is to try and characterize 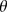 during different time points and study its dynamics. One would expect to find that 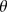 (for excitatory synapses) decreases with time in cases where the neural network has been reported to becomes less sensitive to its input statistics, for example during developmental changes.

## Methods

### “Mean field” Fokker–Planck approach for the learning dynamics

From the synaptic update rule, equation (5), changes in the synaptic weight, 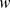, at time 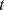, result from either pre or post synaptic firing at time 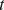, affecting both the depression and potentiation branches (functions) of the adaptation rule. Thus:
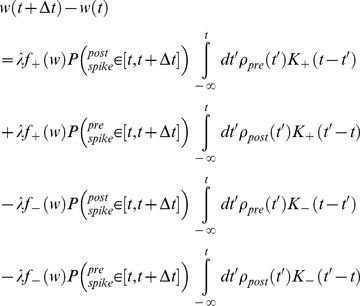
(21)where 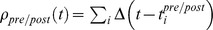 is the firing of the pre/post synaptic cell, as represented by a train of delta functions at the neuron's spike times, with 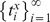 being the spike times, and 
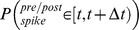
 is 1 if there was a pre/post synaptic spike respectively at the specified time interval 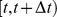 and 0 otherwise.

Taking the short times limit, 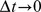: 
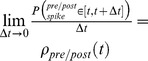
, yields: 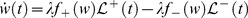
(22)

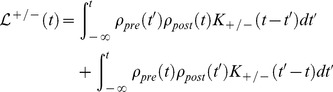
(23)


Assuming the learning process is performed on a much slower time scale than the neuronal dynamics [34], the STDP dynamics samples the pre-post correlations, 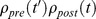, over long periods in which the synaptic weight, 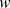, is relatively constant. Using this separation of time scales in the limit of 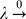, we can approximate 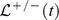 by their time average over period 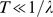. This is the mean-field Fokker-Planck approach to approximating the stochastic dynamics of 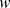. Integration of equation (22) over time yields:
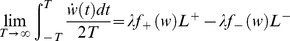
(24)

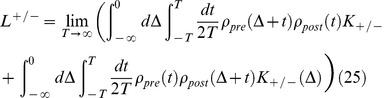
(25)


Assuming we can replace the time averaging of the pre-post correlation with its statistical average for sufficiently large 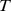,
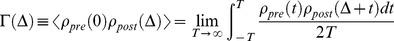
(26)we can substitute equation (26) into equations (25) and obtain the mean field Fokker-Planck equation for the process:
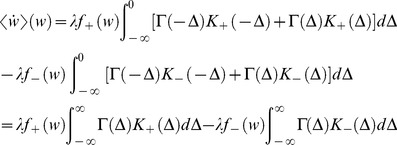
(27)


### Details of the numerical simulations

#### Online supporting information

This manuscript is accompanied by a complete software package that was used throughout the study. This package is a Matlab set of scripts and utilities that includes all the numerical simulations that were used to produce the figures in this manuscript. It also contains all the scripts that generated the figures.

#### The leaky integrate-and-fire model

The learning dynamics of equation (5) was simulated by a single postsynaptic integrate-and-fire cell. As in our previous work [13] the dynamics of the membrane potential of the postsynaptic cell, 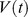, obeys:
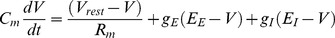
(28)where 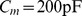 is the membrane capacitance, 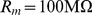 is the membrane resistance, the resting potential is 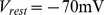, and the reversal potentials are 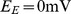 and 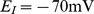. An action potential is generated once the membrane potential crosses the firing threshold 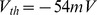, after which the membrane potential is reset to the resting potential without a refractory period. The synaptic conductances, 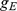 and 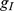, are a superposition of all the synaptic contributions, i.e., each synaptic input is convolved with an α-shaped kernel (that models the filtering nature of the synaptic response) amplified by its synaptic weight and then summed. The terms 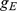 and 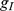 are thus given by:
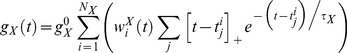
(29)where 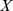 stands for Excitation or Inhibition, 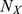 is the number of synapses, 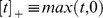 is the dimensionless time value (in seconds), and 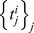 are the spike times of synapse 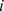. For the temporal characteristic of the α-shape response we chose to use 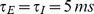, and for the conductance coefficient 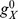 our constant is scaled by 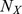 as elaborated below.

In order to estimate the postsynaptic membrane potential in equation (28), the software performs the integration of the synaptic and leak currents using the Euler method with a 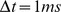 step size. The rationale for using such a low resolution step size and its verification are discussed below.

#### Modeling presynaptic activity

Throughout the simulations in this work, presynaptic activities were modeled by an independent homogeneous Poisson processes, with stationary mean firing rate 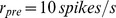. To this end, each of the inputs was approximated by a Bernoulli process generating binary vectors defined over discrete time bins of 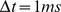. These vectors were then filtered using a discrete convolution α-shaped kernel (as defined above) with a limited length of 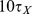 (after which this kernel function is zero for all practical purposes). In all simulations we used: 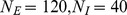.

#### Conductance constants

In order to be compatible with previous studies; e.g., [7], [13], and to have simulations that are executed with a robust and generic software package accompanying this manuscript as File S1, we scaled the synaptic conductance inversely to the number of synaptic inputs in our simulations. We used the following scaling formula 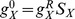, with: 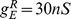, 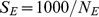, 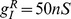 and 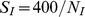, where 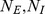 are the number of excitatory and inhibitory presynaptic inputs, respectively.

#### The learning rate

The simulations of the STDP process were carried out to obtain the asymptotic weight distribution of the plastic synapse. Convergence to the asymptotic region was accelerated by manipulating the learning rate constant 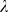 of equation (1). The software code was designed to support a given vector of 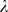 for each minute of the simulation. Specifically we used the following formula to generate this vector: 
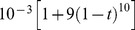
, where 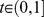, is the ratio between the minute iteration time and the entire simulation time. Examining the behavior of this function shows that it starts from a value of 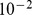 and decays significantly fast, leaving the trailing 70% of the simulation time with more or less the same learning rate of about 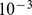.

#### Postsynaptic spike time accuracy vs. simulation step size resolution

Figure 5 shows the remarkable match between the fixed point solution (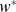) of equation (12), and the asymptotic synaptic weight (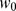) of the simulations; the regression coefficient on the entire set {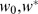} in all the panels is 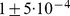 with 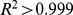 when using an integration step of size 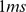. Tests of this kind were performed on simulations using integration steps ranging from 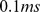 to 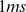 in two calculation modes (see below), and it was found that higher resolution provides a better match to the analytical solution. However, the key feature that contributes to this high degree of similarity between the analysis and the simulations (more than an order of magnitude for the error term 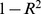) was the definition of the spike times of the postsynaptic cell rather than a 10× decrease of the integration step size.

The spike times of an integrate and fire neuron are defined as the times in which its membrane potential crossed the firing threshold, 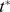. However, in the numerical simulations we used discrete times, 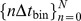. In previous work we define the time of the post-synaptic firing by the last discrete time preceding the threshold-crossing time to: 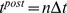 such that 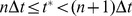. This choice may change the causal order of pre-post firing (from pre before post to simultaneous firing) at time intervals of the time-bin. Consequently, it will affect the STDP rule – mainly when kernels that are discontinuous at zero are used. Here we defined the spike time of the post-synaptic neuron to be: 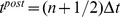 such that 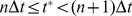 (i.e., shifted by half a time-bin from previous definition); thus, this manipulation retains the causality of firing.

## Supporting Information

File S1 This package (1Syn-STDP4PLOS.zip) is a Matlab set of scripts and utilities that includes all the numerical simulations that were used to produce the figures in this manuscript. It also contains all the scripts that generated the figures. The scripts in the main folder are divided into two categories. The files that begin with “Bat” execute the numerical simulations, and the ones that begin with “Plot” generate the figures. All the supporting numerical utilities are stored in the sub folder “CommonLib”.
(ZIP)
